# The day that belongs to children

**DOI:** 10.1038/s41390-025-04189-5

**Published:** 2025-06-10

**Authors:** Kaan Can Demirbas, Ozgur Kasapcopur

**Affiliations:** 1 Department of Child Health and Diseases, Cerrahpasa Medical Faculty, Istanbul University-Cerrahpasa, Istanbul, Turkey; 2 Department of Child Health and Diseases, Division of Pediatric Rheumatology, Cerrahpasa Medical Faculty, Istanbul University-Cerrahpasa, Istanbul, Turkey

## Abstract

- This article discusses a national holiday in Türkiye, dedicated to children and democracy, and how its celebration in a hospital setting serves as a vital moment of healing and joy for hospitalized children, reaffirming their identity beyond illness and underscoring the societal commitment to protect and celebrate childhood.
- We aimed to reflect this through the lens of a pediatrician to raise awareness and inspire healthcare professionals regarding the emotional nurturing practices in clinical settings.
- It may influence policymakers or hospital administrators to support similar celebratory and therapeutic initiatives, recognizing the role of emotional well-being in pediatric recovery.

## A pediatrician’s reflection on Türkiye’s National Children’s Day

In the heart of Turkey’s national identity lies a day unlike any other: April 23rd – National Sovereignty and Children’s Day. It is a day that bridges democracy and innocence, statehood and joy, the gravity of governance and the lightness of childhood. As a pediatrician practicing in Turkey, this day carries a profound significance. It is more than a commemorative event; it is a yearly reminder that children are not only the future of our nation but also its most vibrant present.^1^

Established by Mustafa Kemal Atatürk, the founder of the Turkish Republic, this unique holiday symbolizes the intimate connection between a free nation’s sovereignty and the well-being of its youngest citizens. Each year, when I walk through the pediatric wards decorated with balloons, hear the laughter of children temporarily freed from the burden of illness, and witness their joy upon receiving simple gifts, I am reminded of the depth of this connection.

A child in a hospital bed is not only separated from health but also from school, friends, playgrounds, and the rhythms of normalcy. They face a disconnection from their developing identity and social network. This emotional toll, though less visible than physical symptoms, is deeply impactful. The hospital is not typically a place one associates with celebration. And yet, every year on April 23rd, something beautiful happens in our pediatric unit. Celebrities arrive to perform for children; volunteers bring toys and books; playgrounds are assembled within hospital corridors. The sterile environment is softened by music, laughter, and vibrant colors. In our own hospital, we take pride in making this day special. Rooms are decorated with streamers and flags, and we hand out symbolic gifts—books, crayons, puzzles—to remind children that even amid illness, they are seen and valued. These moments, while small, hold immense therapeutic power.

As pediatricians, we often measure healing in biomarkers or scans. But there is another kind of healing-emotional, communal, imaginative-those blooms in these April moments. Even if only for a day, children are reminded of who they are outside of their diagnosis: playful, creative, essential. April 23rd serves as a powerful remedy to this disconnection. It reintroduces joy, visibility, and recognition. According to the United Nations Convention on the Rights of the Child, play and leisure are not luxuries but rights. Children deserve spaces to express themselves, to feel joy, and to be recognized for more than their illnesses. National holidays like April 23rd bring these rights to life in public and celebratory ways.

April 23rd is not simply a national holiday. It is a mirror held up to our society’s values — a day where children take center stage in the **democratic narrative**. It reminds us that national strength lies in nurturing joy, and that in celebrating children, we reaffirm our commitment to a future worth building. For us, being pediatricians in a country that honors children so visibly and proudly is a gift. And each year, when we see their smiles brighten a ward, we remember why we chose this path.
